# A pilot factorial randomised cohort trial of manual therapy or acupuncture for low back pain

**DOI:** 10.1186/1745-6215-12-S1-A150

**Published:** 2011-12-13

**Authors:** Vivienne C Dascanio, Yvonne Birks, David Torgerson

**Affiliations:** 1 York Trials Unit, Department of Health Sciences, ARRC Building, University of York, Heslington, York, YO10 5DD, UK

## Background

Randomised control clinical trials of acupuncture have been hampered by the challenges of assessing it as a complex intervention. Controlling for and separating placebo effects whilst identifying its efficacy as a treatment can be difficult [1]. The comparison of acupuncture to other complex interventions has been recommended to assess the effectiveness of acupuncture against other interventions [2].

The objective of this pilot trial is to investigate the feasibility of undertaking a novel randomised cohort design study with a nested factorial RCT, investigating acupuncture alone versus manual therapy alone versus a combination of acupuncture and manual therapy versus usual care.

The pilot will investigate recruitment rates to allow for planning a full-scale trial, identify any compliance issues and strategies for reducing these in a full-scale trial and assess patient’s acceptance and therapist delivery of combined therapies for the treatment of their LBP.

## Methods

The study will follow a randomised cohort trial design and participants from the cohort will be selected to participate in the pilot trial. The use of this design as a recruitment method for nested trials is relatively new methodology but the cohort design has been suggested as an effective method for the use with chronic conditions [3] and its potential for minimising attrition. Attrition is one of the major threats to the internal validity of any trial. The design of this trial specifically reduces that threat [4]. Using a randomised cohort design will provide a ‘run-in period’ of three months, from collecting baseline data to the first set of outcome data. Only participants who return their three monthly questionnaires will be eligible for randomisation to the pilot trial. As the majority of attrition occurs at the first period of follow-up in an RCT, it is expected subsequent attrition, after randomisation, to be minimal [4].

The factorial pilot RCT will investigate the treatment of low back pain with Acupuncture vs Manual Therapy vs Acupuncture and manual therapy vs Usual GP care. All interventions will be delivered by a chartered physiotherapist.

## Results and conclusions

Recruitment and retention rates will be presented. The acceptability and feasibility of the design for use with complex interventions and in a common musculoskeletal condition will be discussed.
